# Draft Genome Sequence of *Mycobacterium heckeshornense* Strain RLE

**DOI:** 10.1128/genomeA.00930-15

**Published:** 2015-08-27

**Authors:** Alexander L. Greninger, Gail Cunningham, Charles Y. Chiu, Steve Miller

**Affiliations:** Department of Laboratory Medicine, UCSF, San Francisco, California, USA

## Abstract

We report here the draft genome sequence of *Mycobacterium heckeshornense* strain RLE isolated from a sputum sample from a patient with shortness of breath. This is the first draft genome sequence of *M. heckeshornense*.

## GENOME ANNOUNCEMENT

*Mycobacterium heckeshornense* is a slowly growing scotochromogenic mycobacterium that was originally isolated in 1994 from a cavitary lung lesion and is named after the Heckeshorn Lung Clinic in Berlin (1). *M. heckeshornense* has since been isolated from a broad range of clinical cases, such as septicemia, osteomyelitis, diskitis, peritonitis, tenosynovitis, and lymphadenitis, as well as from porcine and feline lymph nodes (2–8). It is phylogenetically and phenotypically close to *Mycobacterium xenopi*, which is a common source of confusion and misidentification (1, 5, 9). We sequenced the first draft genome of *M. heckeshornense* isolated from a 2008 sputum sample from a 50-year-old male with HIV and CD4 count of <60 who presented with shortness of breath and cough and was concurrently diagnosed with *Pneumocystis jirovecii* pneumonia.

DNA from *M. heckeshornense* strain RLE was extracted using the Qiagen EZ1 kit, and paired-end libraries were prepared using the Nextera XT DNA library kit, followed by 2 × 80-bp sequencing on Illumina MiSeq. The sequences were adapter and quality (Q20) trimmed using cutadapt, *de novo* assembled using SPAdes version 3.5, metagenomically screened for contaminating sequence with SURPI, and annotated via Prokka version 1.1 (10–13). A total of 8,091,536 paired-end reads were recovered after trimming. *De novo* assembly yielded 191 contigs of >500 bp for a total assembly size of 5,010,173 bp, with an *N*_50_ of 95,235 bp, an average coverage of 126×, and a total of 4,882 coding sequences. Contiguity was interrupted due to the short read length, high G+C content (66%), and presence of many high-copy-number integrases, transposases, and tyrosine recombinases that aligned with between 70 and 85% identity at the nucleotide level to a variety of actinobacteria, such as *Gordonia bronchialis* DSM 43247, *Rhodococcus aetherivorans* strain lcdP1, *Mycobacterium kansasii* 824, *Mycobacterium marinum* strain M, and *Rhodococcus jostii* RHA1.

BLASTn analysis of the complete 16S sequence from strain RLE showed 100% identity to *M. heckeshornense* type strain S369 (NCBI reference sequence NR_028759) and *Mycobacterium* cf. *xenopi* (i.e., a *Mycobacterium* sp. very similar to *Mycobacterium* *xenopi*) strain Hymi_Wue Tb_939/99 (GenBank accession no. AJ243481) and *Mycobacterium sydneyiensis* (GenBank accession no. AF101243), as has been noted for *M. heckeshornense* (14). As no publications are available for these two strains nor have any sequences been deposited, they likely should be renamed as strains of *M. heckeshornense*. BLASTn of the full-length *rpoB* gene revealed 91% nucleotide identity to *rpoB* genes from *Mycobacterium branderi* ATCC 51789, *Mycobacterium celatum* ATCC 51130, *Mycobacterium kyorinense* KUM 060204, and a variety of *Mycobacterium avium* strains, including 104, 2285, and DJO-44271. No high-confidence antibiotic resistance genes were identified by the Comprehensive Antibiotic Resistance Database analysis (15).

### Nucleotide sequence accession numbers.

This whole-genome shotgun project has been deposited at DDBJ/ENA/GenBank under the accession no. LFOF00000000. The assembly described in this paper is the first version, LFOF01000000.
